# Alterations in fecal microbiota composition by probiotic supplementation in healthy adults: a systematic review of randomized controlled trials

**DOI:** 10.1186/s13073-016-0300-5

**Published:** 2016-05-10

**Authors:** Nadja B. Kristensen, Thomas Bryrup, Kristine H. Allin, Trine Nielsen, Tue H. Hansen, Oluf Pedersen

**Affiliations:** The Novo Nordisk Foundation Center for Basic Metabolic Research, Section of Metabolic Genetics, Faculty of Health and Medical Sciences, University of Copenhagen, Universitetsparken 1, 2nd floor, Copenhagen Ø, 2100 Denmark

## Abstract

**Background:**

The effects of probiotic supplementation on fecal microbiota composition in healthy adults have not been well established. We aimed to provide a systematic review of the potential evidence for an effect of probiotic supplementation on the composition of human fecal microbiota as assessed by high-throughput molecular approaches in randomized controlled trials (RCTs) of healthy adults.

**Methods:**

The survey of peer-reviewed papers was performed on 17 August 2015 by a literature search through PubMed, SCOPUS, and ISI Web of Science. Additional papers were identified by checking references of relevant papers. Search terms included healthy adult, probiotic, bifidobacterium, lactobacillus, gut microbiota, fecal microbiota, intestinal microbiota, intervention, and (clinical) trial. RCTs of solely probiotic supplementation and placebo in healthy adults that examined alteration in composition of overall fecal microbiota structure assessed by shotgun metagenomic sequencing, 16S ribosomal RNA sequencing, or phylogenetic microarray methods were included. Independent collection and quality assessment of studies were performed by two authors using predefined criteria including methodological quality assessment of reports of the clinical trials based on revised tools from PRISMA/Cochrane and by the Jadad score.

**Results:**

Seven RCTs investigating the effect of probiotic supplementation on fecal microbiota in healthy adults were identified and included in the present systematic review. The quality of the studies was assessed as medium to high. Still, no effects were observed on the fecal microbiota composition in terms of α-diversity, richness, or evenness in any of the included studies when compared to placebo. Only one study found that probiotic supplementation significantly modified the overall structure of the fecal bacterial community in terms of β-diversity when compared to placebo.

**Conclusions:**

This systematic review of the pertinent literature demonstrates a lack of evidence for an impact of probiotics on fecal microbiota composition in healthy adults. Future studies would benefit from pre-specifying the primary outcome and transparently reporting the results including effect sizes, confidence intervals, and *P* values as well as providing a clear distinction of between-group and within-group comparisons.

## Background

The human gut microbiota refers to the microbes that reside inside the gut and partake in several functions beneficial to the host including fermentation of otherwise indigestible dietary fibers and other food items [1], synthesis of vitamins and amino acids [2], prevention of pathogen colonization [3], maturation and regulation of the immune system [4], modulation of gastrointestinal hormone release, and regulation of brain behavior through bidirectional neuronal signaling as part of the gut-brain axis [5]. The development of culture-indpendent, high-throughput molecular techniques has enabled the identification of previously unknown bacterial species, thereby providing novel insights into the compositional diversity and functional capacity of fecal microbiota. As a result, studies have suggested that disorders such as colorectal cancer, rheumatoid arthritis, type 2 diabetes, and obesity are associated with disease-specific dysbiotic shifts in fecal microbiota [6–11]. Consequently, in recent years the gut microbiota as a potential modifiable risk factor for disease development has received massive attention. One common approach applied to convey health benefits by way of modifying the gut microbiota has been the use of probiotic supplementation. Probiotics are defined as live microorganisms that, when administered in adequate amounts, confer a health benefit on the host in a safe and efficacious manner [12]. Suggested mechanisms by which probiotics may benefit the gut environment and the health of the host include improvement of the intestinal barrier function through effects on the epithelium and mucus lining, production of anti-microbial substances, competition with pathogenic bacteria, and regulation of luminal acidity (reviewed in [13, 14]).

The therapeutic effect of probiotic supplementation has been studied in a broad range of diseases, particularly in regard to gastrointestinal and metabolic disorders where results have supported the potential use of probiotics as therapeutic agents (reviewed in [15, 16]). Common to both sets of disorders is a multitude of readily available, clinically relevant outcome measures (e.g. body mass index, fat mass, insulin resistance, severity of gastrointestinal symptoms) by which to measure treatment effect. The effect of probiotics in disease-free individuals is, however, not as easily assessed. Interpretation of an effect on the composition of fecal microbiota in healthy individuals may be particularly complicated due to the lack of an internationally accepted consensus definition of a normal or a healthy fecal microbial community [17, 18].

Terms such as ecological stability, idealized composition, or favorable functional profile have been suggested as hallmarks of a healthy gut microbiota [17]. These are all very unspecific concepts and the compositional and functional characteristics of a healthy gut microbial community remain to be defined. Furthermore, an effect of probiotics on the composition of the gut microbiota is an intermediate outcome only and should be interpreted with caution in regard to implications for the health of the host. Despite these limitations, several probiotic interventions aiming to observe alterations in fecal microbiota composition have been performed in healthy adults [19–28]. Results from these studies have the potential to provide insights into the underlying mechanisms of probiotics and fecal microbiota. Currently, no systematic review has addressed the effects of probiotics on fecal microbiota composition using high-throughput metagenomic methods (i.e. phylogenetic microarrays, 16S ribosomal RNA (rRNA) sequencing, or shotgun metagenomic sequencing) in healthy adults. In the context of a billion dollar market for probiotic supplements [29] with products being marketed, in part, toward healthy individuals by stating effects on gastrointestinal health, alluding to the fecal microbial community, an overview of the current evidence is warranted.

The objective of the present systematic review was to explore in healthy adults the current evidence for an effect of probiotic supplementation compared to placebo on the composition of human fecal microbiota as assessed by high-throughput molecular approaches in randomized controlled trials (RCTs).

## Methods

We undertook a systematic review of the possible effects of probiotic intervention on the composition of fecal microbiota in healthy adults. The available literature was identified and examined as a systematic review and not a meta-analysis due to the heterogeneity of the study designs and methods. The results are reported in accordance with the PRISMA statement guidelines (Preferred Reporting Items for Systematic Reviews and Meta-Analyses) [30]. The study followed an *a priori* established protocol.

### Eligibility criteria

The criteria for eligibility were healthy adults as study population, probiotics and placebo as intervention, alteration in composition of fecal microbiota assessed by shotgun metagenomic sequencing, 16S rRNA sequencing or phylogenetic microarray methods as the primary outcome, and RCT as the study design with no criteria on study duration. No limits were applied for language or time. Studies not exploiting the randomized controlled design and providing only within-group results (i.e. results before and after the intervention in the probiotic group only) were not included. Moreover, only studies assessing the overall bacterial ecology were included. Accordingly, studies investigating survival of the probiotic strains only were considered ineligible. Studies with interventions combining probiotics with other supplements (e.g. prebiotics, antibiotics, medications) were excluded. If studies had more than two arms, only the comparison of probiotics to placebo was considered. Studies examining both healthy and unhealthy participants were excluded.

### Information sources, search strategy, and study selection

The identification of papers involved four sequential processes performed by two independent reviewers (NBK and TB). On 17 August 2015, a literature search was conducted through multiple electronic databases (PubMed, SCOPUS, and ISI Web of Science) to capture as many relevant citations as possible. The search phrase used was:

Healthy adult AND (probiotic OR bifidobac* OR lactobac*) AND (gut microbio* OR f*cal microbio* OR intestinal microbio*) AND (clinical trial OR intervention OR trial).

In PubMed, “Species” was limited to include only humans and “Article types” was limited to cover “Clinical trial,” “Comparative study,” “Controlled clinical trial,” “Journal article,” and “Randomized controlled trial.”

In ISI Web of Science, “Document types” was limited to contain “Article,” “Clinical trial,” “Other,” and “Abstract.”

In Scopus, “Source type and document type” was limited to comprise “Journals and article,” “Short survey,” and “Erratum.” For “Subject area,” “Agricultural and biological sciences,” “Nursing,” “Pharmacology,” “Toxicology and pharmaceutics,” “Environmental science,” “Veterinary,” “Chemistry,” and “Neuroscience” were excluded.

**Fig. 1:**
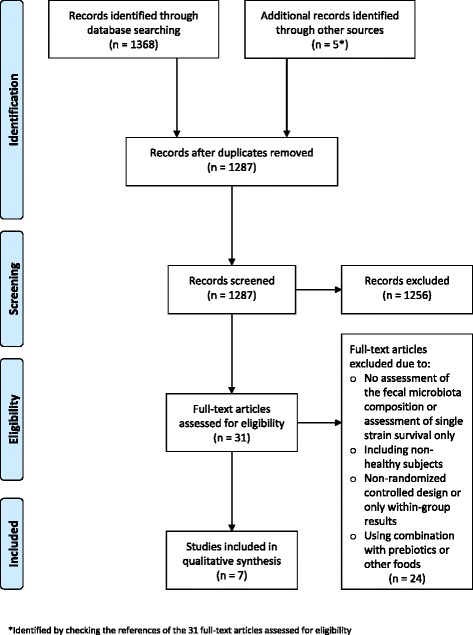
*Flow chart* of literature selection process [30]

Full reports were obtained and screened for all titles appearing to meet the inclusion criteria or in case of any uncertainty. References in 31 full-text articles were also assessed for inclusion in the present review. Screening and eligibility assessment by title and abstract resulted in 1373 citations (Fig. 1). The assessment was performed independently in an unblinded standardized manner by NBK and TB resulting in seven included studies. Any disagreements between reviewers were resolved by consensus.

### Data collection process

Independent data collection was performed by two authors (NBK and TB). Corresponding authors of the following studies were contacted in order to acquire missing information on allocation concealment or other measures of risk of bias: Lahti et al., Rampelli et al., Ferrario et al., Bjerg et al., Hanifi et al., and Simon et al. [19, 21–24, 27]. Unpublished information about blinding was obtained from Lahti et al., Bjerg et al., and Hanifi et al. [19, 21, 22] and the reason for excluding data from three participants from the intervention group (missing fecal samples) was obtained from Hanifi et al. [21].

### Data items

Information extracted from each included RCT was: (1) participant characteristics (including age and gender); (2) intervention (including probiotic strain and dosage as well as mode of administration); (3) design (including study design and duration); and (4) outcome measure (including the microbiomics and intervention effect on overall fecal microbiota structure).

### Quality assessment

The methodological quality assessment of reports of the clinical trials was performed independently by NBK and TB using a three-item instrument (the Jadad score) that evaluates likelihood of bias in research reports [31]. The three items evaluated by a five-point scale are quality of randomization, quality of blinding, and reasons for withdrawal/drop-out (0 = worst, 5 = best). Risk of bias was further assessed regarding concealment of randomization, early termination of trial, blinding of patients, healthcare providers, data collectors and outcome assessors, reporting of drop-out or withdrawal, selective outcome reporting, and other potential biases [32, 33].

### Summary measures

Intervention effects on the overall fecal microbiota structure, that is, richness, abundance, evenness, α-diversity or compositional dissimilarity (β-diversity), were the primary measures of treatment effects.

## Results

### Study selection

A total of 1368 citations were identified through the search in PubMed, SCOPUS, and ISI Web of Science and an additional five were identified through checking the references of relevant papers. After the removal of duplicates, 1287 citations were left. NBK and TB screened the initial search results using abstracts and 1256 citations were excluded as irrelevant for one or more of the following reasons: animal study, meta-analysis/review, non-healthy or non-adult participants, no probiotic intervention, or no assessment of fecal microbiota composition. The full papers of the remaining 31 citations and references therein were assessed to select studies for inclusion using the abovementioned criteria, resulting in the exclusion of 24 studies due to one or a combination of the following reasons: no assessment of fecal microbiota composition, assessment of single-strain survival only, inclusion of non-healthy participants, non-randomized controlled design, provision of only within-group results, and combined intervention of probiotic with prebiotics or other foods. Following the selection process (Fig. 1), seven studies [19–24, 27] remained (five of which were identified by checking the references of relevant papers) and were included in the present systematic review.

### Study characteristics

All seven studies were published in English language journals between February 2013 and October 2015 (EPub June 2015). One study was performed in Finland [22], two in Italy [23, 27], two in Denmark [19, 20], one in the United States [21], and one in Germany [24]. An overview of the study characteristics and main results are presented in Table 1. The studies were designed as RCTs, one of which used a cross-over design [27]. Six studies were double-blinded, whereas one was single-blinded [20]. Participants were all healthy adults (range, 19–88 years) with a proportion of female participants in the range of 50–100 %. The total number of included individuals was in the range of 21–81. The intervention received was probiotics belonging to the genus *Lactobacillus* (n = 5) [19, 20, 22, 24, 27], *Bifidobacterium* and *Lactobacillus* combined (n = 1) [23], or *Bacillus* (n = 1) [21] which was provided in biscuits (n = 1) [23], milk-based drinks (n = 1) [22], sachets (n = 1) [20], or capsules (n = 4) [19, 21, 24, 27] administered at a dose of ~10^9^ to 10^11^ colony-forming units (CFU) for 21–42 days. Three of the studies collected additional samples 1–3 weeks after the intervention had ended [19, 21, 22]. Compliance was assessed by pill count or screening for the probiotic in fecal microbiota and evaluated as sufficient in most of the studies. However, Rampelli et al. found only a trend towards enrichment of the probiotic strain [23]. Habitual diet was assessed in two studies [20, 27]. In the present review, the primary outcome of interest is alterations in fecal microbiota composition, which was assessed by either microarray hybridization (HITchip (n = 1) [22], HTF-Microbi.Array (n = 1) [23]), or next-generation sequencing methods (16S rRNA sequencing on Ion Torrent PGM (n = 1) [27], Illumina MiSeq platforms (n = 1) [24], or 454 pyrosequencing (n = 2) [19, 21]), or metagenomics on a SOLiD 5500×l platform (n = 1) [20]. Of the studies using a 16S rRNA-based approach, one did not report which hypervariable region of the 16S rRNA gene was targeted and no studies targeted the same set of regions. The databases used for mapping the sequences were GreenGenes version 13.5 (n = 1) [27], RDP (MultiClassifier 1.1 or not specified) (n = 2) [19, 24], or both (versions not specified) (n = 1) [21], while two did not report the database used. The study by Brahe et al. [20] used metagenomics and mapped reads to a reference catalogue of 3.3 million bacterial genes [34].

**Table 1:** Characteristics of the studies reviewed

Study	Participant characteristics	Intervention	Design	Microbiomics	Sample size calculation	Probiotic effects on fecal microbiota compared to placebo
Lahti et al. 2013 [22]	25/25 (72 %) 23–55 years Finland	*L. rhamnosus* GG ATCC53103 (1.55 × 10^10^ CFU) in 250 mL milk-based fruit drink	Double-blinded, parallel, two-armed, placebo (drink) controlled (21 days)	16S rRNA (regions V1 and V6); HITchip based characterization of >1000 microbial species-like phylotypes	Post hoc	↔ Composition of the fecal microbiota ↔ Stability of the fecal microbiota quantified by inter-individual and intra-individual correlations within and between time points
Rampelli et al. 2013 [23]	32/32 (59 %) 71–88 years Italy	*B. longum* Bar33 and *L. helveticus* Bar13 (10^9^ CFU) in biscuit	Double-blinded, parallel, two-armed, placebo (biscuit) controlled (30 days)	16S rRNA (region unknown); HTF-Microbi.Array based characterization of 31 phylogenetically related groups	No	↔ Relative abundance of 31 phylogenetically related groups
Ferrario et al. 2014 [27]	22/34* (56 %) 23–55 years Italy	*L. paracasei DG* (>2.4 × 10^10^ CFU) in capsules	Double-blinded, cross-over, placebo (capsules) controlled (two 28-day intervention periods with a 28-day wash-out)	16S rRNA (region V3) sequencing on Ion Torrent platform	No	↔ α-diversity Modified β diversity (with absolute distances higher for the probiotic than for the placebo treatments)
Bjerg et al. 2015 [19]	20/64* (50 %) 20–45 years Denmark	*L. casei* W8® (10^10^ CFU) in capsules	Double-blinded, parallel, two-armed, placebo (capsules) controlled (28 days)	16S rRNA (regions V3 and V4) sequencing on Roche 454 pyrosequencing platform	No	↔ α- and β-diversity
Brahe et al. 2015 [20]	34/58* (100 %) 40–70 years Denmark	*L. paracasei* F19 (9.4 × 10^10^ CFU) or flaxseed mucilage (10 g) in sachets	Single-blinded, parallel, three-armed, placebo (sachets) controlled (42 days)	Quantitative metagenomics on a SOLiD 5500×l platform	Yes	↔ Bacterial gene count (richness) and abundance of specific bacterial genes compared to placebo
Hanifi et al. 2015 [21]	37/81* (52 %) 19–49 years United States	*Bacillus subtilis* R0179 (0.1 × 10^9^, 1.0 × 10^9^, and 10 × 10^9^ CFU, respectively) in capsules	Double-blinded, parallel, four-armed, placebo (capsules) controlled (28 days)	16S rRNA (regions V1 to V3) pyrosequencing	No	↔ β-diversity and OTU based richness
Simon et al. 2015 [24]	21/21 (52 %) 40–65 years Germany	*L. reuteri* SD5865 (2 × 10^10^ viable cells) in capsules	Double-blinded, parallel, two-armed, placebo (capsules) controlled (28 days)	16S rRNA (regions V5 and V6) sequencing on Illumina MiSeq platform	Yes	↔ α- and β-diversity

Participant characteristics are number of participants with microbiome data/number of participants included in the study. Participant characteristics (% women and age range of participants in years) are based upon number of participants included in the study. CFU, colony-forming units. OTU, operational taxonomic unit. ↔ indicates no difference between the probiotic group and placebo. ↑ indicates an increase in the probiotic group compared to placebo. ↓ indicates a decrease in the probiotic group compared to placebo. *P* values are unadjusted for multiple testing. *Performed next-generation sequencing on fecal samples from a subgroup of the included individuals. In the study by Ferrario et al., 22 participants (of 34) completed the study. Bjerg et al. selected 10 (of 32) placebo-treated and 10 (of 32) probiotic-treated participants with the highest triacylglycerol concentration in the blood at week 0. The study by Brahe et al. had a third intervention arm not relevant for the present study where the number of participants were approximately two-thirds of the total number of participants included in the study. Hanifi et al. selected 20 (all) placebo-treated and 17 (of 20) probiotic-treated participants from the group with the highest dose (10 × 10^9^ CFU) of the probiotic treatment

### Risk of bias

Seven studies were identified and evaluated as medium to high quality by the Jadad score (3–5) as presented in Table 2. The quality of the included studies is generally high in regard to risk of bias and the methods of assessing fecal microbiota configuration. However, blinding of healthcare providers, data collectors, and outcome assessors were either not performed or unclearly reported in three of the seven included studies, which may have caused performance and detection bias. Two studies only investigated the effect of the probiotic treatment on a subgroup of participants, which is also a potential source of bias.

**Table 2:** Assessment of risk of bias in the studies reviewed

Study	Concealment of randomization	RCT stopped early	Patients blinded	Healthcare providers blinded	Data collectors blinded	Outcome assessors blinded	Reporting drop-out or withdrawal	Other potential bias	Selective outcome reporting	Jadad score
Lahti et al. 2013 [22]	Yes	No	Yes	Yes	Yes	No	Yes	No	No	4
Rampelli et al. 2013 [23]	Yes	No	Yes	Unclear^a^	Unclear^a^	Unclear^a^	Unclear	Yes^c^	Yes^c^	3
Ferrario et al. 2014 [27]	Yes	No	Yes	Unclear^a^	Unclear^a^	Unclear^a^	Yes	No	No	3
Bjerg et al. 2015 [19]	Yes	No	Yes	Yes	Yes	Yes	Yes	Yes^b^	No	5
Brahe et al. 2015 [20]	Yes	No	Yes	No	No	No	Yes	No	No	4
Hanifi et al. 2015 [21]	Yes	No	Yes	Yes	Yes	Yes	Yes	Yes^b^	No	5
Simon et al. 2015 [24]	Yes	No	Yes	Unclear^a^	Unclear^a^	Unclear^a^	Yes	No	No	4

Based on PRISMA (and Cochrane)’s tools for assessing risk of bias. The Jadad score is a three-item instrument that evaluates likelihood of bias in terms of quality of randomization, quality of blinding, and reasons for withdrawal/drop-out. It is compiled of the following quality items from the table: Concealment of randomization, Patients blinded, Healthcare providers blinded, Data collectors blinded, Outcome assessors blinded, and Reporting drop-out or withdrawal

^a^Double-blinded study but unclear whether healthcare providers, data collectors, and outcome assessors were all blinded

^b^Performed next-generation sequencing on fecal samples from a subgroup of the included individuals. Bjerg et al. selected 10 (of 32) placebo-treated and 10 (of 32) probiotic-treated participants with the highest triacylglycerol concentration in the blood at week 0. Hanifi et al. selected 20 (all) placebo-treated and 17 (of 20) probiotic-treated participants from the group with the highest dose (10 × 10^9^ colony-forming units) of the probiotic treatment

^c^No direct comparison between treatment groups was made for the age-related dysbiosis

### Results of individual studies

In terms of richness, evenness, or α-diversity measures, no effects were observed on the fecal microbiota composition in any of the included studies when compared to placebo and only in the study by Ferrario et al. [27] was it found that probiotic treatment significantly modified the compositional dissimilarity (β-diversity).

In the study by Lahti et al. [22], the temporal stability of fecal microbiota, quantified by the correlation of the fecal microbiota profiles among three time points, did not differ between the probiotic (*L. rhamnosus* GG ATCC53103) and the placebo group.

In the study by Rampelli et al. [23], there was no effect of probiotic supplementation (*B. longum* Bar33 and *L. helveticus* Bar13) on the relative abundance of 31 phylogenetically related groups when compared to placebo. In the same study, the effect of probiotic supplementation on age-related dysbiosis was also evaluated. The probiotic intervention reverted an age-related increase of *Clostridium cluster* Xi, *C. difficile*, *C. perfringens*, *Enterococcus Faecium*, and *Campylobacter* when comparing the probiotic and the placebo group to a common reference of eight young, healthy adults; but no direct comparison was made between treatment groups.

In the study by Ferrario et al. [27], the α-diversity reported as Chao1 and Shannon coefficients and number of detected genera did not change as a result of the probiotic intervention (*L. paracasei* DG) when compared to placebo. Yet, the β-diversity between the probiotic and the placebo group was modified with absolute distances significantly higher for the probiotic than for the placebo treatments when assessed by principal coordinate analysis (PCoA) of weighted UniFrac distances. Accordingly, the relative abundance of Proteobacteria (*P* = 0.006) and Clostridiales genus *Coprococcus* (*P* = 0.009) were increased and the Clostridiales genus *Blautia* (*P* = 0.036) was decreased in the probiotic group when compared to placebo. Additionally, analyses of predicted functional profiles revealed changes in eight Kyoto Encyclopedia of Genes and Genomes modules related to bacterial pathways in membrane transport, amino acid metabolism, energy metabolism, and metabolism of cofactors and vitamins (*P* < 0.05).

In the study by Bjerg et al. [19], the β-diversity was not affected by the probiotic intervention (*L. casei* W8®) compared to placebo when assessed by PCoA of species and genus level Operational Taxonomic Unit (OTU) based UniFrac distances. Furthermore, no difference in α-diversity (Chao1 and Shannon index) or species richness was observed between the probiotic and the placebo group.

In the study by Brahe et al. [20], fecal microbiota was assessed by shotgun-sequencing-based metagenomics. The bacterial gene count (richness) did not change within the probiotic group (*L. paracasei* F19) compared to placebo. Alterations in the abundance of individual bacterial genes (2493 genes assigned to two metagenomic species) were observed after the intervention in the probiotic group. However, fewer alterations were observed in the intervention group compared to the placebo group (7436 genes assigned to six metagenomic species). Yet, no direct comparison between the groups is explicitly stated.

In the study by Hanifi et al. [21], no difference in compositional dissimilarity (β-diversity) between the treatment groups (*Bacillus subtilis* R0179 in different doses) and placebo was shown when analyzed using PCoA based on the UniFrac metric. Sequence reads binned to multiple OTUs assigned to the genus *Ruminococcus* increased in the probiotic group (with the highest dose (10 × 10^9^ CFU, Table 2) compared to placebo (*P* < 0.01).

In the study by Simon et al. [24], the overall composition of fecal microbiota was unaffected by probiotic supplementation (*L. reuteri* SD5865) both in terms of α- (Chao1, Shannon, and Simpson indices) and β- (Bray-Curtis, Morisita-Horn, and weighted UniFrac) diversity.

## Discussion

Overall, this systematic review demonstrates that there is no convincing evidence for consistent effects of probiotics on fecal microbiota composition in healthy adults.

No effects were observed on the fecal microbiota composition in terms of α-diversity, richness, or evenness in any of the included studies when compared to placebo. Only in the paper by Ferrario et al. [27] was it reported that probiotic supplementation significantly modified the overall structure of the fecal bacterial community in terms of compositional dissimilarity (β-diversity) when compared to placebo.

### Study design and reporting of results

Overall, the reporting of the analyses and results was non-transparent and difficult to assess with very few effect sizes, confidence intervals, and *P* values reported. This is possibly due to the fact that fecal microbiomics is a relatively new research area that currently relies heavily on non-parametric statistics and lacks an internationally accepted standard approach of reporting results. Unfortunately, this impedes the comparison of results in the present review. As the only study, Ferrario et al. [27] used a cross-over design, which may not be the ideal design to assess the effects of a probiotic intervention due to the risk of carry-over effects [35]. In the study the probiotic cell count was decreased after the 4-week wash-out period compared with baseline count, suggesting that wash-out was effective. However, a carry-over effect at the outcome level cannot be excluded. Only two studies provided *a priori* sample size calculations [20, 24], of which two calculated statistical power based on alterations in fecal microbiota composition [20, 22]. Thus, several of the studies may well have been underpowered, with an inherent risk of unequal distribution of confounding factors. A potential confounder in the studies reviewed is habitual diet. Human studies have revealed that short-term and long-term changes in diet (such as plant-based vs. animal-based, amount of dietary fibers and fat) influence the fecal microbiota composition and function [36–38]. Hence, the enormous inter-individual variation in the dietary intake and its effect on the fecal microbiota may mask the true picture of the impact of a probiotic treatment. Only one of the included studies monitored habitual diet with the aim of accounting for differences in dietary habits, specifically considering prebiotic fibers, during the intervention period [27]. A major limitation of most included studies is an unclear, inexplicit statement of the pre-specified primary outcome and delimitation of secondary outcomes. Only one study [24] is explicitly labeled as a pilot trial, reporting a multitude of outcomes, only in part addressing multiple testing. Two studies do not address the issue of multiple testing [23, 27], while others report multiple primary outcomes or make no distinction between primary and secondary outcomes [19–22]. Reporting of the results is generally unclear, with between-group comparisons on primary outcomes intermixed with results on secondary outcomes and within-group comparisons of differences between baseline and post-intervention measures.

### Heterogeneity

Although study participants in the included trials were all healthy adults, the demographic makeup varied widely among studies. Rampelli et al. [23] included only elderly individuals, who may respond differently to probiotics than young individuals and Brahe et al. [20] included postmenopausal women only.

Considering that the impact on the fecal microbiota may differ among strains of the same bacterial species [39], despite close phylogenetic relationships, a potential source of heterogeneity is the use of various probiotic agents. Six studies used single-strain interventions with probiotic products belonging to the genera *Lactobacillus* [19, 20, 22, 24, 27] or *Bacillu*s [21]. One study used a double-strain probiotic mixture of bacteria belonging to the genera *Lactobacillus* and *Bifidobacterium* [23]. Whereas the use of different probiotic agents makes it difficult to draw any meta-analytical conclusions, the choice between single-strain and multi-strain intervention is probably of less importance. In most cases, inert bacteria are administered and within a few hours are entering a diverse ecosystem where they are numerically a minority. So while additive or synergistic effects might be observed in vitro, the opportunity for metabolically active strains delivered in combination to result in similar effects in vivo may not present itself.

None of the studies included in the present review comment on the rationale behind their choice of dosage. The International Scientific Association for Probiotics and Prebiotics provides a list of dosages ranging from 1 × 10^8^ to 1.8 × 10^12^ CFU twice daily depending on strain and disease, based on at least one well designed clinical trial showing a beneficial effect for a health promoting or therapeutic outcome [40]. However, the list covers gastrointestinal disorders only and does not address fecal microbiota in healthy participants. In general, different dosages should be assessed to facilitate an interpretation of the dose-response relationship of probiotic consumption on relevant outcomes, rather than on safety and viability alone. The information provided by such studies would enable the identification of the dosage needed to observe an impact on the relevant outcome and add to the likelihood that an observed association is causal [41]. Hanifi et al. [21] examined and detected oral dose-response relationships, but for tolerance and gastrointestinal viability only. As of now, it is impossible to draw any conclusions on the ideal dosage regarding effects on the fecal microbiota composition. Likewise, the optimal duration of intervention remains elusive.

Mode of administration may also contribute to the observed lack of impact on fecal microbiota. Ingested probiotics must survive hostile environments including acidic, protease and bile-salt rich conditions during their passage through the gastrointestinal tract [42, 43]. Currently, screening feces is the only way to assess whether the probiotics have survived through the gastrointestinal tract. Yet, the site of action may be proximal to the colon and it is not necessarily possible to conclude on the degree of colonization or even the amount of bacteria that produce the effect [44]. In contrast to the findings in five of the included studies, the study by Rampelli et al. [23] showed only a trend towards enrichment of the probiotic strain, perhaps due to the use of biscuits as the mode of administration, yet, another reason may be low compliance. This may add to the explanation why little effect of probiotics was found in Rampelli et al. [23]. Compliance was evaluated as sufficient in the remaining studies [19–22, 24, 27].

Further adding to the heterogeneity between studies is the application of different methods for assessing fecal microbiota. Even though all studies applied high-throughput metagenomics approaches, the resolution and specificity levels varied tremendously and no studies used the same methodological approach. Only one study examined the fecal microbiota by an untargeted metagenomic approach using shotgun sequencing and thereby providing information on microbial gene and derived functional levels, free of bias introduced by amplification of a specific genomic region as is the case in targeted 16S rRNA sequencing and array-based analyses [45]. Compositional information can be achieved by mapping the reads to a microbial gene reference catalogue [34], as was done by Brahe et al. [20]. Still, only a fraction of sequencing reads can be mapped to the existing reference catalogues. The targeted 16S rRNA approach provides information at the taxonomic level in the form of abundance and phylogenetic relationship, but the method has pitfalls in PCR amplification steps [46] and cross-platform comparison is not straightforward. Of the included studies, two use 454 pyrosequencing [19, 21], one uses Illumina MiSeq [24], another uses Ion Torrent sequencing [27], and two use phylogenetic microarrays [22, 23]. The sequencing platforms differ in costs, coverage, and length of reads with the Illumina platform becoming more widely used [45]. Community profiles from HITChip correlate well with pyrosequencing-based compositions (Pearson correlations at phylum (average r  =  0.94), class (0.93), order (0.94) and family levels (0.77)) and the HTF microbe.array has demonstrated good reproducibility by cluster analysis of the phylogenetic fingerprint in samples from the same participant [47, 48]. In general, using phylogenic microarray approaches have the advantages of being cost-efficient for compositional characterization; however cross-hybridization may occur and only taxa that are covered by the reference sequences can be detected [47]. Another well-known source of bias in 16S rRNA-based studies is the targeted hypervariable region of the 16S rRNA gene. The region used for analysis in the included studies applying 16S rRNA-based methods varies with one study using V1 and V6 [22], one study using V3 [27], one study using V3 and V4 [19], one study using V5 and V6 [24], and one study does not specify [23]. Several studies have examined the effects of region choice when evaluating fecal microbiota composition with no current international consensus [47, 49–51].

### Probiotics in health and disease

In a recent systematic review including 29 trials studying healthy adults with undisturbed microbiota (using non-high-throughput molecular techniques) only ~20 % showed an effect of probiotic treatment on fecal microbiota. It is concluded that there is little, if any, evidence of an effect of probiotic treatment in circumstances where the microbiota is unperturbed by pathophysiological processes or pharmaceutical treatment (antibiotics or chemotherapy), either concurrent with or prior to intervention. However, where dysbiosis is present or where the microbiota is perturbed, there is some evidence for a restorative or protective effect of certain strains of probiotics, both on the fecal microbial community itself, but more importantly, also on host physiology, e.g. alleviation of gastrointestinal symptoms [18].

In the case of dysbiotic or perturbed microbial communities, any restorative or protective effect on the microbiota alone, without any measurable beneficial effect for the host, would predominantly be of academic interest by improving our understanding of the intestinal ecosystem. In the case of undisturbed microbiota, any inference of health benefit from changes to the microbiota alone would be highly speculative without a direct linkage to relevant host phenotypes. Ideally, hard endpoint data would determine the effects of probiotics in healthy individuals, but considering the time perspective of generating such data this may be long in coming. Until such studies are available, any statement on health benefits of probiotic supplementation in healthy participants would rely on observed effects on biomarkers or other intermediate outcomes.

### Limitations

Limitations of this review include the search terms used to identify relevant papers. In addition to probio*, we specifically searched on bifido* and lacto*, but other search terms such as *Bacillus* and *Saccharomyces* could have been relevant. Publication bias is a well-known challenge within the field of systematic reviews and meta-analyses; however, the majority of the studies included in the present review provide null findings, indicating that this concern may be settled to some extent. Language bias cannot be ruled out since our search was exclusively based on English language dominated sources.

## Conclusions

Based on our review of the available RCTs, we find there is a lack of evidence to conclude whether or not there is an effect of probiotics on fecal microbiota composition in healthy adults, as assessed by high-throughput molecular techniques. A number of issues blur the conclusions that can be drawn from the studies, including small sample sizes with lack of statistical power, low resolution-methods of assessing fecal microbiota composition, inter-individual variation in susceptibility toward the probiotic, use of different probiotic strains either in isolation or in combination, variations in dosage and administration mode of probiotics, duration of intervention, or variation in the habitual diet of participants. Future research on the impact of probiotics on fecal microbiota configuration and function should involve statistically well-powered RCTs in well-phenotyped individuals. Importantly, future studies would also benefit from pre-specifying the primary outcome and transparently reporting the results including effect sizes, confidence intervals, and *P* values as well as providing a clear distinction of within-group and between-group comparisons. For the purpose of demonstrating health benefits of probiotic supplementation, effects should be demonstrated on relevant host phenotypes, which is non-trivial in healthy participants. Studies with microbiome features as the primary outcome should be reserved for improving our understanding of biology in general and our insight into microbial interactions in vivo in particular.

## Abbreviations

CFU: Colony forming units
OTU: Operational taxonomic unit
PCoA: Principal coordinate analysis
RCT: Randomized controlled trial
rRNA: Ribosomal RNA
